# Prader–Willi syndrome: Symptoms and topiramate response in light of genetics

**DOI:** 10.3389/fnins.2023.1126970

**Published:** 2023-02-06

**Authors:** Cécile Louveau, Mimi-Caterina Turtulici, Angèle Consoli, Christine Poitou, Muriel Coupaye, Marie-Odile Krebs, Boris Chaumette, Anton Iftimovici

**Affiliations:** ^1^Centre de Référence pour les Maladies Rares à expression Psychiatrique, GHU Paris Psychiatrie et Neurosciences, Paris, France; ^2^Department of Child and Adolescent Psychiatry, Pitié-Salpêtrière Hospital, Paris, France; ^3^GRC-15, Dimensional Approach of Child and Adolescent Psychotic Episodes, Faculté de Médecine, Sorbonne Université, Paris, France; ^4^Nutrition Department, Rare Diseases Center of Reference “Prader–Willi Syndrome and Obesity With Eating Disorders” (PRADORT), Assistance Publique-Hôpitaux de Paris, Pitié-Salpêtrière Hospital, INSERM, Nutriomics, Sorbonne Université, Paris, France; ^5^Institute of Psychiatry and Neuroscience of Paris (IPNP), INSERM U1266, Université Paris Cité, Paris, France; ^6^Department of Psychiatry, McGill University, Montréal, QC, Canada

**Keywords:** Prader–Willi, topiramate, treatment, genetics, deletion, disomy, personalized medicine

## Abstract

**Introduction:**

Prader–Willi Syndrome (PWS) is a rare genetic condition, which affects one in 25,000 births and results in various phenotypes. It leads to a wide range of metabolic and endocrine disorders including growth delay, hypogonadism, narcolepsy, lack of satiety and compulsive eating, associated with mild to moderate cognitive impairment. Prognosis is especially determined by the complications of obesity (diabetes, cardiorespiratory diseases) and by severe behavioral disorders marked by impulsivity and compulsion. This heterogeneous clinical picture may lead to mis- or delayed diagnosis of comorbidities. Moreover, when diagnosis is made, treatment remains limited, with high interindividual differences in drug response. This may be due to the underlying genetic variability of the syndrome, which can involve several different genetic mutations, notably deletion or uniparental disomy (UPD) in a region of chromosome 15. Here, we propose to determine whether subjects with PWS differ for clinical phenotype and treatment response depending on the underlying genetic anomaly.

**Methods:**

We retrospectively included all 24 PWS patients who were referred to the Reference Center for Rare Psychiatric Disorders (GHU Paris Psychiatrie and Neurosciences) between November 2018 and July 2022, with either deletion (*N* = 8) or disomy (*N* = 16). The following socio-demographic and clinical characteristics were recorded: age, sex, psychiatric and non-psychiatric symptoms, the type of genetic defect, medication and treatment response to topiramate, which was evaluated in terms of eating compulsions and impulsive behaviors. We compared topiramate treatment doses and responses between PWS with deletion and those with disomy. Non-parametric tests were used with random permutations for *p*-value and bootstrap 95% confidence interval computations.

**Results:**

First, we found that disomy was associated with a more severe clinical phenotype than deletion. Second, we observed that topiramate was less effective and less tolerated in disomy, compared to deletion.

**Discussion:**

These results suggest that a pharmacogenomic-based approach may be relevant for the treatment of compulsions in PWS, thus highlighting the importance of personalized medicine for such complex heterogeneous disorders.

## Introduction

Prader–Willi syndrome (PWS) is a rare genetic disease, whose prevalence is estimated at 1/15,000−30,000 worldwide. Typical features may involve a narrow forehead, almond-shaped eyes, thin upper lip and drooping corners of the mouth, as well as very small feet and hands (Cassidy et al., 2012). Their developmental trajectory is marked by severe hypotonia and feeding deficits starting in the neonatal period, and followed by a period of hyperphagia and food obsession, which often leads to severe obesity from childhood to adulthood (Gunay-Aygun et al., 2001; Poitou et al., 2023). It is further complicated by a wide range of endocrine dysfunctions (Tauber and Diene, 2021). Growth hormone deficiency contributes to statural growth delay, an excess of fat mass, and an insufficiency of lean mass, resulting in a decrease in energy expenditure. Hypogonadism may lead to incomplete pubertal development (Noordam et al., 2021). Hypothalamic disturbances may cause aberrant temperature control, while orexin deficits may lead to phenotypes ranging from daytime sleepiness to narcoleptic phenotypes (Colmers and Wevrick, 2013; Kim and Choi, 2013; Beauloye et al., 2015; Grugni et al., 2016; Correa-da-Silva et al., 2021). Insatiable hunger and hyperphagia may be caused by a reduced number of oxytocin neurons in the hypothalamic paraventricular nucleus (Tauber and Hoybye, 2021). Conversely, ghrelin, a hormone secreted by the stomach to stimulate appetite, may be overexpressed (Tauber and Hoybye, 2021). In the absence of a strict diet, weight gain can be very rapid and accounts for much of the morbidity and mortality of these patients (Kim and Choi, 2013; Saunders et al., 2018). Moreover, this food addiction behavior is the main obstacle to autonomy and socialization in patients with PWS, because of clastic crises in connection with dietary frustrations (Salles et al., 2021).

From a neurodevelopmental perspective, learning difficulties, social skills deficits, and severe behavioral problems are important determinants of the functional outcome (Sinnema et al., 2011; Butler et al., 2019). Intellectual deficit is rarely major and is extremely variable from one individual to another. People with PWS may have a generalized anxiety disorder of the obsessive-compulsive type (OCD), including dermatillomania–where scratching lesions can lead to *Staphylococcal aureus* infection, or manual extraction of feces, representing a risk of infectious and gastroenterological complications (Dykens and Shah, 2003; Sinnema et al., 2011; Shriki-Tal et al., 2017; Whittington and Holland, 2018; Guinovart et al., 2019; Tarsimi et al., 2021). The prevalence of psychotic episodes is also increased. Patients may present dysthymic disorders such as a depressive episode, particularly when they are aware of their pathology and its genetic aspect. There are rare reports of bipolar disorder associated with PWS, but this may also be related to the behavioral and hormonal dysfunction of PWS (Bellman et al., 2021). Clinical diagnosis is often challenging for psychiatrists, with overlaps between comorbidities: anxiety disorder, mood disorder, psychotic disorder, personality disorder, autism spectrum disorder, eating disorder, or OCD.

To date, there is no consensus on drug treatment for PWS. However, several studies suggested that topiramate may lead to significant clinical improvement, particularly in cases of compulsive overeating, dermatillomania, and frustration intolerance (Shapira et al., 2002; Smathers et al., 2003; East and Maroney, 2018). Topiramate is an antiepileptic drug classically used to treat generalized and partial epilepsy, migraine headaches, and bipolar disorder, because of its mood stabilizing effect. In PWS, dosages are variable (between 50 and 500 mg/day) and depend mainly on its efficacy and tolerability. The only randomized control trial to date found a significant hyporexigenic action (Consoli et al., 2019). Physiologically, it would seem that topiramate regulates the satiety loop and compulsive behaviors, explaining its effect on appetite and binge eating (Smith et al., 2016; Saunders et al., 2018; Khalil et al., 2019).

Overall, there is great variability both in tolerance and efficacy of topiramate treatment, several studies showing beneficial effects of this treatment, while others warn on side effects (Smith et al., 2016; Consoli et al., 2019; Khalil et al., 2019; Steinhoff et al., 2021). This heterogeneous clinical and treatment response profile may stem from an underlying genetic heterogeneity (Shapira et al., 2002, 2004; Smathers et al., 2003; East and Maroney, 2018; Consoli et al., 2019). Thus, in 60% of cases, there is a 15q11-q13 deletion on the paternal chromosome, while in 40% of cases a maternal uniparental disomy is found, whereby the child has inherited two maternal chromosomes 15. Rarely, imprinting anomalies or translocations are found. Therefore, in this study, we proposed to explore whether the type of genetic anomaly could explain differences in clinical presentation and response to treatment, focusing on topiramate. In a retrospective approach, we tested the difference between the two most frequent anomalies, deletion and disomy, in terms of symptomatology, efficacy, and tolerance of topiramate.

## Materials and methods

### Population

In this monocentric retrospective descriptive observational study, we included all patients having a diagnosis of PWS with genetic confirmation of either disomy or deletion, with no age limit, seen between November 2018 and July 2022 at the reference center for rare diseases with psychiatric expression (“Centre de Références Maladies Rares,” CRMR, GHU Paris Psychiatry and Neurosciences). Most patients were referred to the adult CRMR, from the French reference center for PWS and children CRMR of Pitié-Salpêtrière hospital, as part of a transition from pediatric to adult care.

### Clinical assessment

In a retrospective reading of medical records, the presence of the following clinical information was recorded: age, sex, body mass index (BMI), aggressiveness, anxiety, psychosis (defined by the presence of hallucinations or delusions), depression (according to DSM-5 criteria for major depressive disorder), dermatillomania (according to DSM-5 criteria for excoriating skin disorder). Treatment characteristics were also noted: the use of antidepressant, antipsychotic, or antiepileptic medication, as well as topiramate use, its dosage, and its tolerance and efficacy. The treatment was considered effective when the patient and the family described a stable clinical condition with improvement of the disabling symptoms, impulse control, or weight loss. Tolerability was assessed by the presence or absence of side-effects (hyperammonemia, confusion, increased aggressiveness, sudden weight loss).

### Statistical analysis

Quantitative variables (age, BMI, topiramate dosage) were compared between subjects with deletion and those with disomy with a non-parametric Mann–Whitney–Wilcoxon U test. Categorical variables (presence or absence of symptoms, topiramate tolerance and efficacy) were compared between subjects with deletion and those with disomy with a Chi2-test for proportions. In light of the small number of subjects, we used random permutations to compute a non-parametric *p*-value for each comparison. A conservative significance threshold was set at 0.004 after Bonferroni correction for 12 comparisons, but results below or equal to a nominal significance of 0.05 were also considered in this exploratory study. Lastly, we used a bootstrap simulation to estimate how the difference in variable proportions may vary, providing a non-parametric 95% confidence interval for each distribution. A T-test was used to compare the bootstrapped distributions. Analysis was carried out on Python with SciPy.

## Results

Clinical and therapeutic characteristics of the population are presented in Table 1. There were no differences in age, sex, and BMI between deletion and disomy. In this psychiatric setting, the initial symptoms at first consultation were behavioral disorders with auto- and hetero-aggressiveness.

**TABLE 1:** Clinical and treatment response characteristics.

	Mutation		Statistics	
	**Deletion**	**Disomy**	**U/Chi2**	***P*** **-value**
**Population description**				
Number of subjects	*N* = 8	*N* = 16	–	–
Age (years)	27 ± 6	27 ± 9	29.5	0.56
Sex (F/M)	3/5	5/11	0.00	1.00
BMI (kg/m^2^)	35 ± 12	41 ± 7	16.5	0.66
**Psychiatric symptomatology**				
Aggressiveness	100% (8/8)	100% (16/16)	–	–
Anxiety	100% (8/8)	100% (16/16)	–	–
Psychosis	0% (0/8)	43.75% (7/16)	3.10	0.05
Depression	37.5% (3/8)	100% (16/16)	9.10	0.002
Dermatillomania	37.5% (3/8)	56.25% (9/16)	0.20	0.67
**General medication**				
Antidepressant use	75% (6/8)	62.5% (10/16)	0.02	0.66
Antipsychotic use	62.5% (5/8)	62.5% (10/16)	0.00	1.00
Antiepileptic use	75% (6/8)	75% (12/16)	0.00	1.00
**Topiramate treatment**				
Topiramate use	100% (8/8)	56.25% (9/16)	3.05	0.06
Dose (mg/kg)	1.25 ± 0.73	0.97 ± 0.42	27.0	0.83
Tolerance	100% (8/8)	55.55% (5/9)	2.51	0.08
Efficacy	100% (8/8)	33.33% (3/9)	5.58	0.01

All patients presented with aggressiveness and anxiety. Characteristic depressive episodes of moderate to severe intensity, associated or not with suicidal thoughts, were found significatively higher in subjects with a disomy (100%, 16/16) than in subjects with a deletion (37.5%, 3/8), with a *p*-value of 0.002. Among subjects with disomy, 43.8% (7/16) presented persecutory statements, of intuitive or interpretative mechanism, with partial or total adhesion, while none of the patients with a deletion had psychotic symptoms (*p* = 0.05). All subjects had eating disorders of the bulimic type with a compulsive mechanism. Among compulsive behaviors, dermatillomania, mainly on the forearms, thorax and neck, with infected scratch marks in some cases, was found in 37.5% (3/8) of patients with deletion and in 56.3% (9/16) of patients with disomy (*p* = 0.67). Two subjects with disomy reported compulsions to remove feces resulting in lesions of the anal margin.

There was no difference between the two groups regarding antidepressant, antipsychotic, or antiepileptic use. In cases when topiramate was introduced, it was with an initial dosage of 25 mg/day, increased by 25 mg/day every 7 days, after a weekly clinical assessment for efficacy and tolerance. The maximum dosage used was 200 mg/day, with no difference in dosage per kg between groups. There was a tendency to a smaller proportion of subjects receiving topiramate in the disomy group (*p* = 0.06), as well as a tendency of lower efficacy (*p* = 0.01), and tolerance (*p* = 0.08) in this group compared to the deletion group. The main side effects of treatment were hyperammonemia, clinically observed as confusion and temporo-spatial disorientation with increased behavioral disturbances, and biologically confirmed by plasma assay. This poor clinical tolerance of topiramate led to its discontinuation. Thus, none of the patients with disomy were completely stabilized clinically, with persistent behavioral disorders such as intolerance to frustration and aggressiveness (albeit less than at first assessment).

**FIGURE 1:**
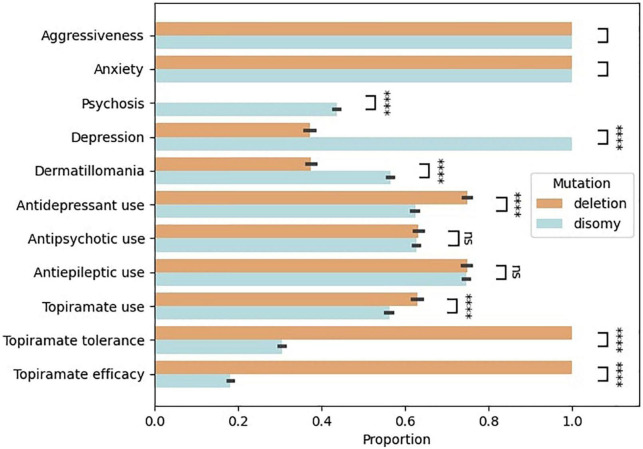
Clinical profile of patients with Prader–Willi syndrome depending on the genetic mutation (deletion versus disomy). Bootstrap simulation based on the 24 patients of the cohort. A *T*-test was used to compare the bootstrapped distributions. **** Simulated *p*-value < 0.0001.

The bootstrap simulation of the variation in proportion of symptoms and treatment response between groups is shown in Figure 1. It suggests that patients with disomy tend to have more severe psychiatric symptomatology in terms of psychosis, depression, and dermatillomania (simulated *p*-value < 0.0001). Likewise, patients with disomy tend to receive more antidepressants (simulated *p*-value < 0.0001). There tends to be more use of topiramate in subjects with deletion than disomy, and less tolerance and efficacy in subjects with disomy than deletion (simulated *p*-value < 0.0001).

## Discussion

In this retrospective analysis of subjects with Prader-Willi syndrome, we studied differences in clinical presentation and treatment response between subjects with 15q11 deletion and those with a uniparental maternal disomy. Our current results suggest a clinical profile dependent on the genetic mutation. Disomy seems to lead to a more pronounced psychiatric symptomatology, with more psychotic and dysthymic disorders than in the deletion group. Disomy may also be associated with less tolerance (hyperammonemia, drowsiness, depressive syndrome) and lower response of impulsive behaviors to topiramate treatment, whereas topiramate seems more effective and better tolerated in subjects with a deletion. This genotype-dependent difference therefore requires a cautious monitoring that may benefit from a more personalized approach. In the disomy group, antidepressant treatments were less prescribed while depression was more frequently reported than in the deletion group. This could be explained by the fact that patients with disomy are more at risk of pharmaco-induced manic episodes (Sinnema et al., 2011). It also explains the general use of antiepileptic treatment (75% of patients), including topiramate, which provides thymic coverage. It should be noted that among the antipsychotic treatments prescribed in this cohort, aripiprazole represented a treatment of choice. Indeed, a recent study highlighted the benefit of aripiprazole treatment in clastic seizures (Deest et al., 2022). Moreover, it has the added advantage of having less metabolic side effects (weight gain), making it the antipsychotic treatment of choice in this condition (Gupta et al., 2021; Sobiś et al., 2022).

However, the size of the cohort is small and does not allow definitive conclusions. To increase the size of the cohort, we aim at extending collaborations between CRMRs in a multicentric study. Importantly, it should be noted that all patients were referred to the CRMR because of severe psychiatric symptoms. This referral constitutes a selection bias that may explain the higher proportion of disomy and lower tolerance of treatment. Interestingly, while in the literature, disomy accounts for only 25−30% of the genetic anomalies found in PWS, here it represented 66% of the population. This tentatively supports the idea of a more severe psychiatric expression in this subgroup, in line with previous reports suggesting a higher risk of psychotic disorders in disomies (Aman et al., 2018; Butler et al., 2019). Of note, we excluded one patient with an imprinting mutation from the analysis. Clinically, his profile was more similar to patients with disomy. The patient had suicidal thoughts and psychotic symptoms. He was treated with an antiepileptic and an antidepressant.

This specific recruitment of patients with severe behavioral difficulties may also explain the observed intolerance to topiramate in a number of subjects, which contrasts with the recent randomized trial of topiramate, reporting a good overall tolerance (Consoli et al., 2019). Another limit of our analysis is its retrospective design that did not allow us to report specific levels of hyperammonemia in relation to topiramate dosage. The results presented here are therefore preliminary and invite further study. The prospective collection of new data will allow the study to gain in power.

From a cognitive perspective, there is no clear difference between disomy and deletion. No difference in overall intelligence quotient (IQ) was reported between these groups. Performance IQ was higher in those with a deletion, while verbal IQ was higher in those with a disomy, who also were reported to have poorer visual acuity and stereoscopic vision (Postel-Vinay et al., 2006). This lack of difference in cognitive profiles may be due to higher heterogeneity inside each group. In subjects with disomy, cognitive function seems more preserved in the case of uniparental disomy, heterodisomy being more favorable than isodisomy (Zhang et al., 2022). In subjects with deletion, phenotype varies according to the length of the deletion. Of the two main deletion types, the long and the short forms, depending on the break points on chromosome 15, the longer form is accompanied by a more marked clinical picture (Butler et al., 2004; Milner et al., 2005; Varela et al., 2005). Unfortunately, due to the retrospective nature of the study, we did not manage to obtain specific deletion types with known break points for each patient. This heterogeneity at the level of the genetic anomaly calls for more specific genotype-based phenotyping (hetero- versus iso-disomy, and long versus short form of deletion).

In conclusion, Prader–Willi syndrome is highly heterogeneous both at clinical and genetic levels and may benefit from a genetically-based phenotyping to identify specific profiles. Should our results be replicated in a larger cohort, it would suggest that the type of mutation (disomy or deletion) could be a genetic marker of response to topiramate treatment.

## Data availability statement

The raw data supporting the conclusions of this article will be made available by the authors, without undue reservation.

## Ethics statement

Ethical review and approval was not required for the study on human participants in accordance with the local legislation and institutional requirements. The patients/participants provided their written informed consent to participate in this study.

## Author contributions

CL, BC, and AI contributed to study design. CL and M-CT contributed to data gathering. CL and AI contributed to data analysis. CL, M-CT, AC, CP, MO-K, BC, and AI contributed to data interpretation and manuscript writing. All authors contributed to the article and approved the submitted version.
