# Corrigendum: Prader-Willi syndrome: Symptoms and topiramate response in light of genetics

**DOI:** 10.3389/fnins.2023.1189154

**Published:** 2023-03-31

**Authors:** Cécile Louveau, Mimi-Caterina Turtulici, Angèle Consoli, Christine Poitou, Muriel Coupaye, Marie-Odile Krebs, Boris Chaumette, Anton Iftimovici

**Affiliations:** ^1^Centre de Référence pour les Maladies Rares à expression Psychiatrique, GHU Paris Psychiatrie et Neurosciences, Paris, France; ^2^Department of Child and Adolescent Psychiatry, Pitié-Salpêtrière Hospital, Paris, France; ^3^GRC-15, Dimensional Approach of Child and Adolescent Psychotic Episodes, Faculté de Médecine, Sorbonne Université, Paris, France; ^4^Nutrition Department, Rare Diseases Center of Reference “Prader–Willi Syndrome and Obesity With Eating Disorders” (PRADORT), Assistance Publique-Hôpitaux de Paris, Pitié-Salpêtrière Hospital, INSERM, Nutriomics, Sorbonne Université, Paris, France; ^5^Institute of Psychiatry and Neuroscience of Paris (IPNP), INSERM U1266, Université Paris Cité, Paris, France; ^6^Department of Psychiatry, McGill University, Montréal, QC, Canada

**Keywords:** Prader–Willi, topiramate, treatment, genetics, deletion, disomy, personalized medicine

In the published article, an author name was incorrectly written as Turtuluci. The correct spelling is Turtulici.

The authors apologize for this error and state that this does not change the scientific conclusions of the article in any way. The original article has been updated.

